# Exploring Perinatal Asphyxia by Metabolomics

**DOI:** 10.3390/metabo10040141

**Published:** 2020-04-04

**Authors:** Emanuela Locci, Giovanni Bazzano, Roberto Demontis, Alberto Chighine, Vassilios Fanos, Ernesto d’Aloja

**Affiliations:** 1 Department of Medical Sciences and Public Health, University of Cagliari, 09042 Monserrato (CA), Italy; bazzanog@gmail.com (G.B.); demrob@unica.it (R.D.); al.chighine@gmail.com (A.C.); ernestodaloja@gmail.com (E.d.A.); 2 Department of Surgical Sciences, University of Cagliari, 09042 Monserrato (CA), Italy; vafanos@tin.it

**Keywords:** perinatal asphyxia, hypoxic-ischemic encephalopathy, ^1^H NMR, GC-MS, LC-MS, metabolomics

## Abstract

Brain damage related to perinatal asphyxia is the second cause of neuro-disability worldwide. Its incidence was estimated in 2010 as 8.5 cases per 1000 live births worldwide, with no further recent improvement even in more industrialized countries. If so, hypoxic-ischemic encephalopathy is still an issue of global health concern. It is thought that a consistent number of cases may be avoided, and its sequelae may be preventable by a prompt and efficient physical and therapeutic treatment. The lack of early, reliable, and specific biomarkers has up to now hampered a more effective use of hypothermia, which represents the only validated therapy for this condition. The urge to unravel the biological modifications underlying perinatal asphyxia and hypoxic-ischemic encephalopathy needs new diagnostic and therapeutic tools. Metabolomics for its own features is a powerful approach that may help for the identification of specific metabolic profiles related to the pathological mechanism and foreseeable outcome. The metabolomic profiles of animal and human infants exposed to perinatal asphyxia or developing hypoxic-ischemic encephalopathy have so far been investigated by means of ^1^H nuclear magnetic resonance spectroscopy and mass spectrometry coupled with gas or liquid chromatography, leading to the identification of promising metabolomic signatures. In this work, an extensive review of the relevant literature was performed.

## 1. Introduction

Perinatal asphyxia (PA) and neonatal encephalopathy (NE) are well-known causes of neonatal morbidity and mortality worldwide. Whenever a hypoxic-ischemic event in the peripartum period may be identified as a putative/certain biological cause, the clinical condition is referred as hypoxic-ischemic encephalopathy (HIE).

Despite a huge improvement being observed in the availability of new and efficacious diagnostic and therapeutic tools in gynecologists’ and pediatricians’ paraphernalia in the last decades, neurological and intellectual disabilities still represent an unbearable burden for families and societies in nearly 25% of individuals with a mild Sarnat grade of HIE [1]. Since 2010, experts have highlighted the need for improvements in adjuvant therapies to hypothermia and in the identification and validation of biomarkers of both damage and outcome to reach the best protocol to protect the infant’s brain and reduce the disabilities related to such a condition [2].

### 1.1. Pathophysiology of Perinatal HIE

HIE is a direct and an indirect consequence of both abrupt and prolonged deprivation of systemic oxygen and reduced cerebral blood flow [3]. Hypoxic-ischemic modifications in the full-term infant trigger several changes at molecular and cellular levels, which may end in cell death and in local/systemic inflammation. The shortage of oxygen, which acts as final electron acceptor in the electron transport chain (ETC) during aerobic respiration, induced by hypoxia and by ischemia, boosts reactive oxygen species (ROS) generation at the cellular level. Generated ROS attack surrounds components at both the mitochondrial and cellular level, leading to mitochondrial dysfunction and permanent damage to cells. The pathogenesis of HIE is strongly influenced by the failure of several potent fetal compensatory mechanisms to cope with the ‘physiological’ hypoxia during pregnancy and delivery. The final clinical outcome of such an insult is a wide spectrum of neurological deficits, ranging from behavioral and motor impairments to general developmental delays to seizures related to structural brain damage.

The severity of the clinical picture of HIE infants is the final result of an uneven combination of several factors, and among them the length and strength of hypoxic insult, together with fetal metabolic conditions before the hypoxia onset. For this reason, the pathological effects are complex to forecast, and they evolve over time. They may be related to two main pathological phases: A primary and a secondary energy failure. Primary energy failure is the first biological effect of both hypoxia and a reduction of cerebral blood flow and it mainly takes place before birth. While the impairment of blood flow is responsible for the progressive reduction of glucose availability needed to fuel brain cells’ metabolism and survival, the concurrent depletion of oxygen content in the blood leads to significantly less energetic availability and to increased lactate production [4]. As a whole, a failure in maintaining the cerebral basal metabolism, which is highly glucose demanding, is achieved and a generalized, although topographically well-defined, hypoxic-ischemic sufferance arises and stays.

At a higher and more knotty level, the lower oxygen content in blood triggers generalized compensatory mechanisms, such as the blood flow’s redistribution to vital organs. The newborn brain reacts to this evolving hypoxic-ischemic condition by producing ROS and releasing excitatory molecules, mainly glutamate (whose release may activate several receptors, primarily N-methyl-D-aspartic acid, MDA ones), allowing an influx of intracellular calcium, chloride, and sodium together with water into cerebral cells [5]. As a result, a complex scenario, caused by inflammation, brain cell damage, and blood–brain barrier (BBB) dysfunction, takes place. The extent and diffusion of the primary energy failure is responsible for the secondary damage appearing, after a brief recovery period (taught to be the best therapeutic window), once blood flow is restored. The secondary failure from an energetic point of view occurs in a temporal window ranging from 6 to 48 h after the onset of hypoxia-ischemia. The pathogenesis of the related brain damage is not fully understood, but it seems to be due to the perseverance of inflammation, oxidative stress, and excitotoxicity. The newborn brain seems to own several features, such as a low concentration of antioxidants, high need of oxygen, higher concentration of unsaturated fatty acids, and easier release of free iron (Fe^2+^) from proteins reacting with peroxides to form more free radicals, which make the brain more prone to permanent damage whenever a critical point of energy deprivation is reached [6]. To avoid or to reduce all or some of these damaging mechanisms, few therapeutic strategies are available at the moment.

### 1.2. Therapeutic Strategies for HIE

Despite the clearer pathological scenario that has been achieved in the last years (either by animal models or by human clinical trials), therapeutic hypothermia (TH) initiated within 6 h after birth for moderate or severe HIE is up to now the only established and standardized treatment, with its efficacy having been proven by many randomized clinical trials [7,8,9,10,11]. The temporal threshold value of 6 h, as for the full-term newborn indication for treatment, is under scrutiny, but no conclusive assessment has been reached up to now.

TH-protective effects are thought to be related to its ability in reducing ROS and glutamate levels, slowing down cerebral oxygen demand, and limiting apoptosis [12]. Neuroprotective agents are, in principle, mandatory in a 360-degrees therapy, but none are currently approved as a gold standard in the clinical management of hypoxic-ischemic babies. This apparent failure seems to be related to the knowledge gap in the exact single mechanism of action or in their combination (in their synergetic effects with TH). Animal models of neuroprotective agents for primary energy failure, such as isoflurane, desflurane, and sevoflurane, are all based on preconditioning efforts to adapt cerebral tissues to a falling concentration of blood oxygen [13,14]. All the approaches based on the reduction of primary energy failure seem to not be feasible in a human context at the moment, although they are very useful regarding the knowledge of the underlying mechanisms of cellular/tissue damage.

The experimental evidence from animal models [15] suggests an involvement of erythropoietin (EPO) receptors during the first hours after hypoxic-ischemic insult. It has been postulated that since EPO receptors are expressed nearby the site of injury, EPO may play a neuroprotective role by reducing the primary brain edema secondary to glutamate toxicity [16] and the volume of the ischemic area [17]. Limited EPO human trials, although promising, failed to show a significant reduction of infant deaths, but a subtle decline of the risk of disability at 18 months, especially in moderate HIE, was observed [18].

Another experimental treatment to be used as delayed therapy is hematopoietic stem cell transplant from the umbilical cord blood. Some clinical trials are underway in HIE and cerebral palsy contexts, with this approach able to be used not only in the strict time window proposed for TH but even in a longer period. The suggested protective mechanism of stem cell therapy operates at several levels, being able to modulate/interact with inflammation, apoptosis, and oxidative stress, but also enhancing regeneration [19]. 

Several drugs (such as melatonin, magnesium sulphate, topiramate, allopurinol, and xenon) have so far been proposed, alone or in combination with TH, and different mechanisms of action have been hypothesized, mainly based on their properties as anti-apoptotic, anti-inflammation, and antioxidant agents. None of these drugs have shown a sound therapeutic advantage, so cannot be considered as mandatory in the treatment of moderate/severe HIE, even though results were encouraging and they are thus worthy of further investigation [20].

### 1.3. Looking for Biomarkers

Nowadays, scientific attention is devoted to the development of new tools for the early diagnosis of PA, the identification of the severity of the brain injury, and the prediction of short- and long-term outcomes, in order to choose the most appropriate neuroprotective treatment to reduce the death rate, neurologic sequelae, and multiorgan failure (MOF) onset. Despite the efforts devoted to the study of different possible indicators of PA or HIE via the alterations of inflammatory proteins, neuron-specific proteins, metabolite pathways, microRNAs, and others, until now, no single biomarker has been validated for diagnosis or to predict the outcome [21]. A good overview of the newly available blood biomarkers for HIE newborns’ outcome prediction has recently been published; however, once again, the authors concluded that the use of multiple biomarkers rather than single ones is likely to be the most accurate way to identify the injury and predict its severity and final outcome [22]. More recently, Magnetic Resonance Imaging (MRI) within 2 weeks after birth coupled with multi-channel Electroencephalography (EEG) and amplitude-integrated EEG was shown to have good sensitivity and specificity for the prediction of unfavorable neurological outcomes [23]. Furthermore, coagulation tests showed a promising and very high predictive value on HIE grade, seizures, and mortality [24]. Despite these markers potentially helping in improving the risk stratification of permanent neurological impairments, all of them are focused on the anatomic–functional results of the hypoxic-ischemic insult more than on the dynamic modification of the metabolism at the cerebral level. The current need is to define a profile or a set of biomarkers that could be used in a narrower temporal window to help clinicians in early neuroprotection.

In the last years, much attention has been devoted to the use of metabolomics, a multiparametric approach for the simultaneous identification and quantification of low molecular weight metabolites present in a biological fluid or tissue and for monitoring the modifications induced by a pathological condition. Metabolomics, giving a holistic view of the metabolic status of an organism, seems to be a powerful platform for unravelling a polymorphic syndrome, such as PA. Indeed, with respect to the use of a single marker, the possibility of measuring the whole metabolomic profile and the screening of molecular intermediates of different biological pathways give a multiparametric response that appears to be more adequate for facing any complex and multifactorial disease.

Moreover, the study of the dynamic alterations of metabolomic profiles can help to improve different crucial clinical aspects, including early PA diagnosis, differentiation of PA versus HIE, response to pharmacological or TH treatments, and, possibly, the prediction of early and late neurodevelopmental outcomes.

Metabolomics studies based on the use of nuclear magnetic resonance (NMR) spectroscopy and mass spectrometry (MS) coupled with gas or liquid chromatography (GC or LC) have been performed to investigate PA in both animal models and humans. Different biological fluids have been analyzed in both untargeted and targeted ways to obtain metabolic fingerprints or to study selected sets of key metabolites. Once a metabolomic profile or a set of specific biomarkers is validated, future clinical implications could be the development of appropriate kits for the metabolomic analysis of newborns’ biofluids to be used at the bedside for early diagnosis and outcome prediction. Among the latter, the possibility for use in the field of neonatology urinary samples to monitor the time-related modifications of a metabolomic profile may guarantee an early and non-invasive harvesting of biofluids (first post-birth urine), a precocious insight into the severity of the hypoxic insult and into the possible outcome, and a snapshot of metabolic modifications prior, during, and after the TH. In this work, we present a review of all the existing literature on the use of metabolomics applied to the study of PA. Major characteristics, concerning the design of the experiment, the sampling methods, the analytical platforms, and the relevant results, are summarized.

## 2. Results and Discussion

In the last 20 years, almost 30 scientific papers dealing with metabolomics and PA have appeared in the literature. These metabolomics studies were mainly performed on animal models, due to the very low incidence of this disease and to ethical reasons, although some studies on human newborns have been performed. Different biofluids and tissues were analyzed in murine, swine, ovine, and non-human primate models. Each biological matrix gives a different snapshot of the metabolic status of the individual under study; blood and CSF metabolomic modifications provide real-time monitoring of the acute hypoxic condition, while urinary modifications are delayed and depend on the renal function and the individual clearance of the metabolites. Nonetheless, urine is the only biofluid that can be safely (and ethically) withdrawn in human newborns. The only other biofluid analyzed in human studies is cord blood.

Biological specimens were analyzed by NMR spectroscopy and GC- or LC-MS spectrometry, and, in a few cases, multiple analytical platforms were used. Each of these platforms has its own advantages and drawbacks. ^1^H NMR is non-destructive, fast, reproducible, and requires minimal sample preparation. It is non-selective, allowing the simultaneous detection of a large number of metabolites present in a sample in one experiment. However, compared to GC- and LC-MS, which can be used to detect metabolites in the picomolar range of concentration, NMR lacks sensitivity, and can be used to detect metabolites only in the micromole range. Due to the fact that the information gained by the use of these analytical tools is complementary, a combination of these techniques is the best approach to analyze the largest number of metabolic features.

In the next paragraphs, the metabolomics literature devoted to PA and HIE is described. The works devoted to the study of animal models are treated separately from those performed on human infants. A brief summary of each study is also reported in Table 1 and Table 2, where the study population, comparison groups, biofluid of choice, analytical technique, and major metabolite modifications are listed.

### 2.1. Animal Models

In animal models, biofluids, such as urine, plasma, cord blood, and cerebrospinal fluid (CSF), and tissues, such as brain, retina, and choroid, were analyzed by NMR and GC- or LC-MS.

In 2001, Van Cappellen Van Walsum et al. investigated the effects of mild and severe hypoxia on near-term fetal lamb CSF by ^1^H NMR [25]. Fetal hypoxia was achieved by decreasing the maternal FiO_2_, leading to acidosis. A paramedian abdominal incision was made followed by hysterotomy. The fetal head was extracted, and an arterial catheter was placed. In the severe hypoxia group, an arterial pH of 7.1 was achieved; in the mild hypoxia group, an arterial pH of 7.23–7.27 was reached and maintained for 2 h. Control baseline samples were also collected. CSF was withdrawn from the fetal cisterna magna through a midline incision in the skin of the neck dorsal surface. Compared to the baseline CSF values, severe hypoxia led to an increase of choline, lactate, alanine, phenylalanine, tyrosine, lysine, branched chain amino acids (BCAAs), and hypoxanthine, which were also found to be increased at the end of the 2-h mild hypoxia period. While choline was correlated to possible cell membrane damage, the other metabolic alterations were related to energy failure in the fetal lamb brain. The increase of free amino acids was ascribed to the degeneration of nerve cells injured by hypoxia. Interestingly, the authors found elevated CSF levels of 3-hydroxybutyric acid and BCAA during severe hypoxia and suggested their use as alternative substrates for energy production in fetal hypoxic brains, whereas the same was not found during mild hypoxia.

Almost 10 years later, Liu et al. extensively investigated murine models of neonatal asphyxia, focusing on the NMR analysis of brain tissues [26,27,28,29]. In particular, after inducing hypoxia-ischemia in neonatal mice and rats, they tried to identify the metabolomic signatures associated to the brain injury, distinguishing normothermic from mild TH recoveries. Most of these experiments were performed ex vivo on neonatal rat cerebrocortical slices [26,27,28]. Asphyxia was simulated via oxygen-glucose-deprivation (OGD) and different thermal treatments were applied for recovery: normothermia (37 °C), hypothermia (32 °C immediately after OGD), and delayed hypothermia (32 °C, 15 min after OGD). Tissue slices were extracted and analyzed via multinuclear (^1^H, ^13^C, and ^31^P) NMR. Different levels of ATP, ADP, phosphocreatine, acetate, adenine, alanine, choline, isoleucine, lactate, leucine, tyrosine, and valine were found in mild hypothermic versus delayed and normothermic treatments. In a following study, brain perchloric acid extracts of mice who underwent right carotid occlusion, 30 min of hypoxia-ischemia, and 3.5 h of either hypothermia (31 °C) or normothermia (37 °C) 7 days after birth were analyzed by ^1^H NMR spectroscopy [29]. Several metabolites were found to distinguish the hypoxic-ischemic group versus the treatment groups, but due to the paucity of the samples, only unsupervised Principal Component Analysis (PCA) models were built comparing all the different groups together, and no predictive multivariate models could be built. Globally, these studies indicated hypoxia-related alterations associated to the energy metabolism and suggested glial integrity as a key component of the neuroprotective effect of mild hypothermia.

Several research papers were devoted to the study of the effect of asphyxia and resuscitation on the plasma metabolome of newborn piglets [30,31,32,33,34,35]. Solberg et al. investigated the effect of hypoxemia of different durations (40–140 min) and resuscitation with different oxygenation regimens (100% oxygen, 100% followed by room air, or solely room air) in the plasma of newborn piglets by targeted LC-MS [30]. Plasma metabolites were found to be significantly modified after hypoxia. Interestingly, while lactate was not correlated with the duration of hypoxia, the ratios of alanine and glycine to BCAA (Ala/BCAA and Gly/BCAA) showed a very good correlation with the length of hypoxic insult. Plasma metabolites’ modifications following resuscitation were highly dependent on the resuscitation protocol used. TCA cycle intermediates (succinate, fumarate, and α-ketoglutarate) and acylcarnitines, which were accumulated during hypoxia, markedly decreased with the room air regimen, suggesting an earlier recovery of the mitochondrial function, while the 100% oxygen treatment was associated with a slower cellular metabolic recovery. In a following study, the same piglet model was investigated via an untargeted liquid chromatography-time of flight mass spectrometry (LC-TOFMS) to evaluate the plasma metabolomic modifications associated with intense hypoxia and reoxygenation with room air [31]. After the induction of severe hypoxia (53.4 ± 17 min FiO_2_ 0.08), plasma choline, fatty acids, hypoxanthine, and other intermediates of purine and pyrimidine metabolism were increased with respect to control samples, while approximately 2 h after room air reoxygenation, the metabolomic fingerprint was almost recovered and comparable to that observed before the hypoxic insult. On the basis of these results, the authors in a following study proposed a metabolite score to estimate the duration and intensity of hypoxia. The score, based on the relative intensities of three plasma metabolites (choline, 6,8-dihyroxypurine, and hypoxanthine), was shown to be highly correlated with the hypoxia time and to have a better predictive ability at 2 h after resuscitation compared to lactate, which is currently considered as the gold standard [32]. Furthermore, the use of plasmatic choline and related metabolites was proposed for grading the intensity and duration of tissue hypoxia, which correlates with the degree of brain damage [33]. Solberg et al. further investigated the effects of profound hypoxia on the retinal tissue in the previously well-established hypoxic-ischemic neonatal piglet model [34]. Targeted ultra-performance liquid chromatography-mass spectrometry (UPLC-MS/MS) allowed the identification of cytidine-di-phosphate-choline (CDP-choline), an intermediate compound of phosphatidyl-choline biosynthesis, as a candidate biomarker directly correlated to retinal hypoxia. The simultaneous study of both retinal and choroid tissues indicated distinct metabolomic responses to hypoxia, suggesting regional sensitivity to this insult [35].

Another series of papers were devoted to the study of urinary metabolomics’ modifications induced by hypoxia in newborn piglets [36,37,38,39]. Atzori et al. proposed the use of ^1^H NMR metabolomics to characterize the urinary profiles of newborn hypoxic-reoxygenated piglets [36]. After hypoxic insult, piglets were resuscitated using different reoxygenation protocols, with oxygen concentrations ranging from 18% to 100%. Despite the different treatments, the authors analyzed all the piglets as a whole, comparing the urinary profiles at baseline, before hypoxia, and at the end of the experiment, indicating a group of discriminant metabolites. Moreover, the authors focused their attention on the profiles of seven piglets who did not respond to resuscitation and died, comparing their basal profiles with those of piglets who showed a short recovery time without asystole. A supervised Partial Least Squares-Discriminant Analysis (PLS-DA) model was built for the classification of the non-surviving animals, and discriminant metabolites were indicated but not discussed. In a following paper, the same authors showed that the piglets resuscitated with low oxygen concentrations (18% and 21%) had a shorter recovery time, while those resuscitated with high concentrations (40% and 100%) showed a longer recovery time [37]. They built a model comparing these two classes of animals and found a profile related to the different oxygen concentration treatments dispensed to the animals. In particular, glucose was higher in the high oxygen concentration group, whereas acetoacetate, alanine, succinate, dimethylamine, methanol, N-phenylacetylglycine, and sarcosine were found to be increased in the low oxygen concentration group. The authors further confirmed their results, indicating that room air resuscitation was related to shorter recovery times and higher survival rates [38]. Skappak et al. identified by ^1^H NMR metabolomics a set of 13 urinary metabolites that allowed hypoxic piglets to be distinguished from normoxic controls [39]. These metabolites (1-methylnicotinamide, 2-oxoglutarate, alanine, asparagine, betaine, citrate, creatine, fumarate, hippurate, lactate, N-acetylglycine, N-carbamoyl-b-alanine, and valine) were mainly involved in cellular energy metabolism and amino acid deregulation following hypoxia. Using an independent set of samples not included in the original model, the authors showed that no single metabolite could be used to classify the animals, whereas the set of the 13 selected metabolites correctly classified hypoxic samples with a 90% accuracy.

Sachse and coworkers examined ^1^H NMR plasma and urine metabolomic profiles in a cohort of 125 newborn pigs asphyxiated until asystole and resuscitated with different cardiopulmonary resuscitation protocols, with the aim of characterizing the response to the different protocols, predicting outcomes, and correlating the metabolic modifications between the two biofluids [40]. Despite the severe alterations caused by asphyxial cardiac arrest, the direct comparison of the metabolic profiles originating from the two biological matrices showed only a poor correlation. The authors concluded that urine is not the ideal biofluid for real-time monitoring of acute conditions, such as hypoxia, as the urinary metabolome modifications are delayed with respect to blood alterations and dependent on the individual renal function. Blood modifications were almost completely recovered after resuscitation, but no differences were observed in response to the different resuscitation protocols.

Juul et al. extensively explored PA in a non-human primate model, namely *Macaca Nemestrina*, using two-dimensional gas chromatography with time-of-flight mass spectrometry (GC×GC-TOFMS) [41,42,43]. In their first work, asphyxia was induced in utero by clamping the umbilical cord blood for 15 or 18 min before delivery [41]. Pre-asphyxia and post-asphyxia umbilical artery cord blood samples were collected from 20 animals and compared with pre- and post-delivery samples of 5 animals who did not undergo umbilical cord occlusion (sham group). Ten metabolites were identified in the post-asphyxia metabolomic profile, with succinate and malate modifications being the most relevant. The latter two respectively showed a 60- and a 6-fold concentration increase with respect to the pre-asphyxia samples. No direct comparison between the asphyxia and the sham group was performed at the two timepoints. In a following study, the authors used the same model, withdrawing four arterial blood sample at defined timepoints (5 min, 24, 48, and 72 h) after delivery [42]. Moreover, the asphyxiated newborns were randomized to three treatments groups: The first no treatment, the second standard TH, and the third standard TH plus EPO. Alterations of circulating metabolites were found and compared among the different neuroprotective treatment groups. Citrate, succinate, fumarate, and malate were all highly increased after asphyxia, confirming their previous results that identified succinate and malate as the most altered one. If so, they have been proposed as potential biomarkers, even more sensitive than lactate in the human context, for early diagnosis of birth asphyxia. A set of eight metabolites were correlated to early and long-term neurodevelopmental outcomes, and four of them, namely citrate, fumarate, lactate, and propionate, were also correlated with death or cerebral palsy. In their most recent study, the authors modified their model, including some postnatal hypoxic episodes in order to mimic apneic events that occur in human newborns with HIE [43]. A protocol of intermittent hypoxia from 3 to 8 times a day for 3 days was applied. Plasma metabolomics together with magnetic resonance imaging (MRI) scans were performed. A transmission electron microscopy (TEM) analysis was carried out on brain tissue samples after necroscopy. Patterns of injury in the basal ganglia, thalamus, and brain stem were observed in both MRI and TEM images and a tentative correlation with few metabolites was proposed.

As a whole, data concerning animal models of PA are very heterogeneous and the designs of the experiments are not comparable at all in terms of the biofluid of choice, primary and secondary endpoints, and causative method of hypoxia/ischemia.

A common drawback in all the animal models is the translatability of the obtained metabolomic pattern to humans. The insult suffered by the newborns of the different species—lack of available oxygen to maintain the basal activities of cells, organs, and apparatus of the body—is so ‘basic’ that it allows inference on a ‘shared’ biological response across the different species. The asphyxia metabolome is the final collection of molecules coming from all the pathways activated by the living beings to struggle against the abrupt/ongoing shortage of oxygen, and the response to such a noxa is mainly based on the shift, or the coexistence, of aerobic and anaerobic glycolysis. If so, it is possible to hypothesize that the relevant metabolomic profile may share all, or the vast majority, of the metabolites in all living beings. If this issue may result in an advantage in this scenario, it represents, at the same time, a limit to the specificity of the abovementioned results. It is assumed that the metabolomic profile related to hypoxia-ischemia is highly specific because in a multivariate statistical approach it is clearly distinguishable from the one obtained by healthy controls or by the one pertaining to asphyxiated newborns without any clinical signs of early impairment. To challenge the real discriminative power of the assumed ‘PA/HIE profile’, this should be compared with profiles obtained by newborns affected by other diseases that may mimic a hypoxic and/or ischemic damaging mechanism (e.g., neonatal sepsis, hemorrhagic shock, mitochondrial congenital diseases, and so on).

In the animal models, the choice of the ‘ideal’ biological fluid is without any restriction, with cord blood, blood, cerebral spinal fluid, urine, and slices of fixed tissues having been investigated. The results of these experiments are highly interesting to unravel the basic mechanism of the damage at cellular and organ levels, but their use in the human context is hampered by the difficulty in replicating them in human experimental setups.

In Table 1 and Table 3, the main results and the shared metabolites are summarized. The need for a common animal protocol is of paramount importance to enhance the efforts to understand the damaging mechanism and to optimize the therapeutic approaches to prevent or to reduce the risk of permanent impairment in infants.

### 2.2. Human Studies

Only few studies have addressed the metabolomic alterations occurring in human neonates after a hypoxic/ischemic insult or associated to HIE. This is mainly due to the low incidence of this disease and to the difficulty of sampling newborns for ethical reasons. Because of that, the majority of the human newborn studies are focused on the urinary metabolome, with this specimen being the biofluid of choice due to the possibility of collecting it longitudinally and non-invasively.

In 2006, Chu and coauthors were the first to demonstrate the potential use of metabolomics for the study of urinary profiles of newborns with clinical evidences of asphyxia and consequent neurodevelopmental sequelae [44]. GC-MS was performed on urine samples collected from a cohort of 256 neonates in the first 12 h after birth and on a second sample collected between 12 and 48 h. Among these, only 24 (9.4% of the cohort) required immediate post-delivery admission to the intensive care unit; 13 out of 24 asphyxiated newborns showed a good outcome, whereas the remaining 11 showed a poor outcome (3 died and 8 were diagnosed for HIE and developed seizures within 7 days from delivery). Eight organic acids, related to energy and oxidative stress metabolism, discriminated HIE asphyxiated versus non-HIE asphyxiated and 13 matched control newborns. The authors attributed to this subset of urinary biomarkers a highly prognostic value related to the worst outcome.

Almost 10 years later, Longini et al. identified a panel of altered ^1^H NMR urinary metabolites in 6 asphyctic preterm newborns compared to 8 matched controls [45]. They collected samples in a non-specified moment during the first 2 days of life. They identified metabolomic alterations in the ^1^H NMR urinary profile of the asphyctic group and showed that the profile did not recover the physiological status after 24–48 h from resuscitation.

A longitudinal study of the urinary metabolome of 12 HIE-diagnosed full term newborns was performed by GC-MS [46] and ^1^H NMR [47]. According to the local guidelines, these neonates underwent TH starting in the first 6 h and maintained this during 72 h after the hypoxic-ischemic event. Urine samples were collected at predefined timepoints, i.e., immediately after birth, before starting TH, at the end of the TH treatment, and at 1 month after birth. For the ^1^H NMR study, urine samples were also collected from matched healthy newborns at birth and used as control samples. The HIE urinary profile at birth was identified and compared with each subsequent collection timepoint. The results indicated dynamic changes over time in the HIE urinary profile from birth to 1 month of life, reflecting either the effects of the TH treatment or the physiological growth of the newborns. TCA cycle intermediates (citrate, α-ketoglutarate, cis-aconitate, and succinate), pyruvate, DMA, DMG, lactose, and galactose increased in HIE newborns during the first month of life, whereas lactate, myoinositol, hypoxanthine, choline/phosphocholine, arginine, and MNA decreased over time. These modifications were mainly related to the derangement of energy cellular metabolism and alteration of homeostasis. Remarkably, the urinary profile of the three babies who eventually died during the first week of life could be distinguished, already at birth, from those of the surviving HIE babies. Indeed, significantly higher amounts of urinary lactate were found in HIE non-surviving babies’ samples at birth, while the lactate levels of the surviving HIE babies were comparable to those of the healthy controls at birth. At 1 month of life, the urinary metabolome of the HIE babies was significantly modified with respect to birth, and was closer, even though still distinguishable from, to the profile of the healthy newborns at birth.

Walsh et al. performed the first serum metabolomic study on full-term newborns with PA using a targeted liquid chromatography-tandem mass spectrometry (LC-MS/MS) approach on umbilical cord blood samples [48]. Three groups of individuals were enrolled: HIE-diagnosed infants with quantification of the brain injury severity based on the Sarnat score, asphyxiated infants without HIE, and matched healthy controls. Major alterations following hypoxia were observed in 29 metabolites, including amino acids, acylcarnitines, and phospholipids. Compared to the matched controls, 14 metabolites were found to be significantly altered in asphyxiated newborns, independently of the HIE occurrence. However, more interestingly, nine metabolites differed only in the HIE samples versus controls, and six differed in the asphyxia versus controls. Moreover, by applying a logistic regression model based on five selected metabolites, the authors could classify the severity of HIE with high specificity and sensitivity. However, the model was not validated in an independent cohort as it is commonly recommended. In a following study, the authors used ^1^H NMR to further explore their PA cohort, with a focus on the central energy metabolites that were not previously quantified [49]. They found 12 metabolites increased in both asphyxia and HIE versus matched controls, 6 additional metabolites increased solely in asphyxia without HIE, and only one metabolite, methionine, significantly increased in the HIE group exclusively. Alanine, choline, creatinine, glycerol, lactate, and succinate were directly correlated to the HIE severity. The accumulation of succinate in severe HIE babies was related to the possible role of Hypoxia Inducible Factor 1-alpha (HIF-1α) in mediating the neurological injury. Acetone and 3-hydroxybutyrate increased in asphyxiated but decreased in severe HIE babies. This indicates that asphyxiated infants retain the capacity to oxidize fatty acids, producing ketone bodies that provide an alternative energy source for the brain, whereas this capacity is reduced in severe HIE babies who are not able to produce neuroprotective ketones. The cohort was longitudinally monitored for some years, until the authors showed that a metabolite score based on the neonatal alterations of succinate, glycerol, O-phosphocholine, and 3-hydroxybutyrate performed quite well for the prediction of the neurodevelopmental outcome of HIE infants at 3–4 years of life [50].

In 2017, Sánchez-Illana and coauthors reported on a randomized, multicenter, double-blinded, placebo-control trial, aiming at the evaluation of the neuroprotective effect of topiramate (TPM) in addition to moderate total body TH in full-term HIE diagnosed newborns. In this context, 61 HIE individuals were longitudinally evaluated and compared with 19 matched healthy controls, due to the unavailability, for ethical reasons, of sample groups of HIE babies not undergoing TH [51]. In contrast to the previously described studies, where the attention was mainly devoted to the assessment of specific metabolomic profiles related to the asphyxia condition and the identification of potential biomarkers related to the severity of the injury, the present work was focused on the study of the modifications of selected key energy-related metabolites in response to the TH. HIE newborns’ plasma samples were collected at birth (umbilical cord blood or, if not available, the first extracted blood sample) at 24, 48, and 72 h after the first TPM dose and compared with 19 blood samples of healthy controls collected at a time ranging from birth to the third day of life. All samples were submitted to GC-MS analysis. Eight metabolites, comprising lactate, pyruvate, ketone bodies (acetoacetate and 3-hydroxybutyrate), and intermediates of the TCA cycle (succinate, fumarate, malate, and a-ketoglutarate), were quantified in the different timepoint samples. Provided that the comparison of the metabolite concentrations of babies treated with TPM or placebo did not show any significant difference, the results indicated that after the hypoxic insult, higher levels of lactate and pyruvate, and lower levels of acetoacetate and 3-hydroxybutyrate were found in HIE plasma samples compared to control ones. Surprisingly, the measured levels of all the TCA cycle intermediates did not show statistically significant modifications in comparison to controls. During the 72 h of the TH treatment, acetoacetate, succinate, fumarate, and α-ketoglutarate remained constant, while a significant decrease in lactate, pyruvate, and 3-hydroxybutyrate, and a significant increase in malate were observed. The results were interpreted as a partial recovery of the aerobic metabolism.

El-Farghali et al. quantified the amino acids and acylcarnitines in cord blood dried spots collected from 45 full-term newborns exposed to PA (16 HIE and 29 non-HIE) and 20 matched healthy controls using UPLC-MS [52]. They detected differences in asphyctic samples with respect to controls but could also distinguish asphyxia from HIE. Moreover, they analyzed the differences between survivors and non-survivors. The authors pointed out the possibility of using these metabolite alterations as early bedside biomarkers for differential diagnosis and for the prediction of short-term outcomes.

To summarize the results, even in the human context, the high variability in the designs of experiments, primary and secondary endpoints, drawing time pace, and adjuvant therapies hinders a clear understanding of the clinical scenario. Despite a more restricted availability of analyzed biofluids (mainly urine and cord blood), the primary endpoints are interspersed in the research of biomarkers of damage and outcome, with the latter being further divided into early and late neurodevelopmental ones.

All the research groups involved in the use of a metabolomic approach to PA seem tightly linked to their starting point of view of the phenomenon, not focusing on a wider picture of the clinical scenario. Nowadays, as there are several other available tools (neuroimaging, aEEG or multichannel EEG, coagulation profiles, or circulating biomarkers) that are useful to stratify the risk of a permanent impairment, the main effort of the metabolomics community should be, in our personal opinion, focused on the identification of an early metabolomic profile able to support the clinical decision of TH, shared by all the infants with the clinical signs required by international guidelines, and adjuvant therapies, which can be personalized on the basis of the relevant metabolomics profile, to be used in monitoring the biological evolution (not the clinical one) in the first week of life.

There is a compelling need for a longitudinal, multicenter, prospective study starting from the metabolite score on cord blood proposed by Ahearne et al. [50] and embracing a day-by-day metabolomics profile focusing on lactate, pyruvate, alanine, BCAA, ketone bodies, TCA intermediates (succinate, malate, fumarate, α-ketoglutarate), purine metabolites, and choline, as the ‘fil rouge’ metabolites linked to the recovery of a ‘physiological’ status.

### 2.3. Limits and Pitfalls

Although there is an urgent need endorsed by all the scientific and clinical communities to screen infants with PA at risk of a clinical encephalopathy in the very first hours after birth, the goal of identifying a metabolomic profile associated to the PA or HIE is not accomplished yet [53].

This is related to the multifactorial nature of this pathological condition that makes it difficult to compare the different studies reported in the literature. The biofluid of choice, sampling time, cause of PA, timing of PA occurrence and its duration, and analytical procedures are the most diversified. In human studies, major limitations are related to the scant number of enrollable patients per center, the lack of an experimental arm with untreated (no TH) PA control groups, and the uneven choice of biofluids and tissues. The urge to promote multicentric studies is growing, although treatment practices for infants undergoing TH are very different from one center to the other, with protocols only partially overlapping [54]. In clinical metabolomics studies, every difference in therapeutic or in assistance protocols may display peculiar metabolic signs in the metabolome, acting as confounding elements and deserving an analytical focus. Only a stringent design of the experiment and a tight adherence to the protocol may guarantee reproducible results to be implemented in the routine treatment of these patients.

Nevertheless, some common alterations have been found representing a common thread, a fil rouge, to be used to roughly discriminate affected from healthy children. As expected, because of the metabolic shift form aerobic to anaerobic glycolysis due to the shortage of oxygen, an increase of lactate is universally described after the hypoxic event in all the analyzed biofluids of both animal models and humans, with a trend toward normalization after reoxygenation or TH treatment. Similarly, a surge of succinate, a commonly recognized marker of ischemia-hypoxia, is described in blood samples and brain tissues but not in urine samples. The increase of this metabolite in blood plasma is directly related to the hypoxic insult, more than to the ischemic one where no flow is observed in the injured area, as it is the mitochondrial response to cope with the reduction of oxygen as a final electron acceptor at ETC [55]. In an anoxic-hypoxic milieu, fumarate acts as an electron acceptor and it is reduced to succinate by the reverse activity of succinate dehydrogenase, causing the accumulation of this metabolite, with α-ketoglutarate dehydrogenase being the limiting enzyme in this mitochondrial safeguard mechanism. The usefulness of succinate as marker of hypoxia-ischemia in circulating biofluids is limited by the high degree of inter-individual variability recently described in an animal model [56] and by the very short half-life of succinate in blood (with an average 5-min re-oxygenation time after a severe ischemic-hypoxic insult being sufficient to restore the baseline values in blood and in the ischemic heart) [57].

Fumarate and malate, which together with succinate are TCA cycle intermediates, increased in all the biofluids, including urines. Quite common behaviors can be found in the increase of hypoxanthine, alanine, BCAA, choline, osmolytes such as myoinositol and betaine, and 3-hydroxybutyrate. Some promising metabolite index scores, obtained by the conjunct use of potential biomarkers’ concentration ratios, have been proposed as being significantly related to the injury or the outcome, but until now, they have not been validated in an independent cohort of patients.

## 3. Conclusions

With the advent of ‘omics’ techniques, the main interest of both biological and clinical research has rapidly moved from the ‘ideal biomarker’ of damage/outcome to a biological signature/phenotype related to the injury’s biological network. Although the chase of a set of biomarkers or of a metabolomics phenotype seems to be promising, the goal is still ‘elusive’ [21,53]. Nowadays, as it is possible to analyze the hypoxic-ischemic insult, together with its direct/ indirect effects, from several perspectives, a holistic approach to this clinical conundrum seems the best choice to unravel the complexity of the biological cascade blooming from the original noxa.

As a matter of fact, the huge amount of genomics, transcriptomics, and metabolomics data so far published have not been able to generate a validated profile or a set of biomarkers to be implemented in everyday clinical routine. Several explanations may be found for this apparent inconclusiveness. Firstly, the clinical picture known as HIE may be different at the origin but convergent at the final pathological mechanisms. Secondly, the experimental approach in the newborn context is hindered by ethical and legal boundaries, as it is sometimes not acceptable to conduct a randomized controlled trial (e.g., TH in mild HIE) or even experiment with a placebo arm. Thirdly, the biological specimens and sampling time employed in the polymorphic experimental settings are different from one study to another. If so, the reproducibility and comparability of results fade away. Finally, the population under investigation is generally small, and multicenter investigations are difficult to organize due to the relatively high variability in pharmacological approaches to sustain the vital functions of newborns.

The need for the right profile, from the right biological samples, at the right time, and in the right patient is still under scrutiny.

## Figures and Tables

**Table 1:** Metabolomics studies performed on animal models.

Study	Population	Groups	Animal	Sample	Method	Biomarkers Findings
Van Cappellen Van Walsum (2001) [25]	23	Severe hypoxia vs. controls	Lamb	CSF	^1^H NMR	↑ Lactate, hypoxanthine, alanine, 3-hydroxybutyrate, choline, phenylalanine, tyrosine, lysine, BCAA ↓ Glucose
		Mild hypoxia vs. controls				↑ Lactate, hypoxanthine, alanine, phenylalanine, tyrosine, lysine, BCAA
Liu (2011) [26]	10	Hypothermic treatment vs. normothermic treatment	Rat	Brain tissue slices	^1^H NMR ^31^P NMR	↑ ATP, ADP, phosphocreatine, N-acetylaspartate, glutamate, taurine ↓ Acetate, adenine, alanine, choline, isoleucine, lactate, leucine, tyrosine, valine, inosine, arginine
Liu (2012, 2013) [27,28]	10	Hypothermic treatment vs. normothermic treatment	Rat	Brain tissue slices	^1^H NMR ^13^C NMR ^31^P NMR	↑ Taurine, phosphocreatine, glutamine
Liu (2013) [29]	10	Hypoxia vs. other treatments	Mice	Brain tissue	^1^H NMR	↑ Alanine, ADP, choline, lactate, succinate, valine, γ-aminobutyrate, isoleucine ↓ ATP, phosphocreatine, phosphocholine, malate, aspartate, taurine, N-acetylaspartate
		Hypothermic treatment vs. other treatments				↑ Taurine, histidine, malate, ascorbate ↓ Fumarate, succinate, glutamine, isoleucine, N-acetylaspartylglutamate, acetate, formate
Solberg (2010) [30]	33	Hypoxia vs. baseline	Piglet	Plasma	FIA-MS/MS LC-MS/MS	↑ Fumarate, succinate, lactate, α-ketoglutarate, long-chain acylcarnitines, alanine, BCAA ↓ Glutamate, citrulline, free carnitine, decadienyl-L-carnitine
		Reoxygenation vs. hypoxia				↑ Glutamate, citrulline ↓ Fumarate, succinate, lactate, α-ketoglutarate, long-chain acylcarnitines, lysine, leucine, isoleucine
		Duration of hypoxia				↑ Alanine/BCAA, glycine/BCAA
Solberg (2016) [31]	32	Hypoxia vs. controls	Piglet	Plasma	LC-TOFMS	↑ Choline, hypoxanthine, 6,8-dihydroxypurine, cytidine, uridine, glycocholic acid, guanine, uric acid, inosine, BCAA, long chain acylcarnitines, glutamine
		Reoxygenation (room air)				Return to baseline levels
Kuligowski (2017) [32]	32	Duration of hypoxia	Piglet	Plasma	LC-TOFMS	Score index based on choline, 6,8-dihydroxypurine and hypoxanthine compared to lactate
Sánchez-Illana (2017) [33]	32	Hypoxia vs. controls	Piglet	Plasma	LC-MS/MS	↑ Choline, cytidine, uridine, lactate
		Reoxygenation vs. controls				↓ Choline, cytidine, uridine
Solberg (2013) [34]	10	Hypoxia vs. controls	Piglet	Retinal tissue	UPLC-QTOF-MS UPLC-MS/MS	↑ Cytidine diphosphate-choline, pyroglutamic acid, GSSG ↓ Cytidine diphosphate-diacylglycerol
Arduini (2014) [35]	10	Hypoxia in retina vs. controls	Piglet	Retinal and choroid tissues	UPLC-MS/MS	↓ Glucose-6P, pyruvate, isocitrate, α-ketoglutarate, malate
		Hypoxia in choroid vs. controls				↑ Lactate
Atzori (2010) [36]	40	Baseline vs. resuscitation with different oxygen concentration	Piglet	Urine	^1^H NMR	Urea, malonate, creatinine, methylguanidine and hydroxyisobutyrate
Murgia (2013) [37]	40	Resuscitation with lower oxygen concentration vs. resuscitation with higher oxygen concentration	Piglet	Urine	^1^H NMR	↑ Acetoacetate, alanine, succinate, dimethylamine, methanol, N-phenylacetylglycine, sarcosine ↓ Glucose
Fanos (2014) [38]	40	Reoxygenation at 21% oxygen vs. baseline	Piglet	Urine	^1^H NMR	↑ Glucose, alanine, lactate, 3-hydroxymethyl glutarate, succinate, malonate ↓ Creatinine, sarcosine, glutamine, acetoacetate, phenylalanine, hippurate, trimethylamine, pyruvate
		Reoxygenation at 18/21% oxygen vs. baseline				↑ Glucose, lactate, alanine, glycerate, pyruvate, malonate, glycine, succinate, 3-methyladenine, acetylglycine, glutaconic acid, 4-hydroxyphenyl pyruvate, 3-hydroxymethyl glutarate
		Reoxygenation at 40/100% oxygen vs. baseline				↑ Creatinine, urea, citrate, tartrate, ethanol, glucose, indoxyl sulfate
Skappak (2013) [39]	32	Hypoxia vs. controls	Piglet	Urine	^1^H NMR	↑ 1-Methylnicotinamide, 2-oxoglutarate, alanine, asparagine, betaine, citrate, creatine, fumarate, lactate, N-acetylglycine, N-carbamoyl-β-alanine, valine ↓ Hippurate
Sachse (2016) [40]	125	2 h after ROSC vs. baseline	Piglet	Plasma	^1^H NMR	↑ Succinate, fumarate, pyruvate, malate, lactate, glucose, choline, creatinine, hypoxanthine, acetate, alanine, glutamine, glycine, myoinositol
		4 h vs. 2 h after ROSC				↑ Choline, creatinine, acetate ↓ Succinate, fumarate, pyruvate, lactate, glucose, hypoxanthine, alanine, glycine
		2 h after ROSC vs. baseline		Urine		↑ Succinate, fumarate, lactate, glucose, choline, creatinine, hypoxanthine, alanine, glycine, leucine, valine ↓ Creatinine, formate, 3-hydroxyisovalerate
		4 h vs. 2 hours h ROSC				↑ Glucose, alanine, leucine, valine ↓ Fumarate, formate, creatinine
Beckstrom (2011) [41]	25	Post-hypoxia vs. pre-hypoxia	Macaque	Cord blood plasma	GC×GC-TOFMS	↑ Succinate, malate, lactate, glutamate, 9H-purine, glycerol, glucose-1P, arachidonate, leucine, creatinine, fructose, myoinositol, butanoate, pantothenate
		Hypoxia vs. sham group				↑ Succinate, malate, lactate, glycerol, arachidonate, creatinine, fructose, glucose-1P, butanoate, pantothenate
Chun (2015) [42]	33	TH/TH+EPO (different time points) vs. untreated	Macaque	Cord blood plasma	GC×GC-TOFMS	Aminomalonate, arachidonate, butanoate, citrate, fumarate, glutamate, lactate, malate, maltose, myoinositol, propanoate, succinate
McAdams (2017) [43]	4	After resuscitation vs. baseline	Macaque	Cord blood plasma	GC×GC-TOFMS	↑ Arachidonate, fumarate, succinate, propanoate, myoinositol
		24 h after resuscitation vs. baseline				↑ Myoinositol, glutamate, choline, glycine, serine, oleate, erytro-pentonate

ADP, adenosine diphosphate; ATP, adenosine triphosphate; BCAA, branched chain amino acids; EPO, erythropoietin; FIA-MS/MS, flow injection analysis-tandem mass spectrometry; GC×GC/MS, two-dimensional gas chromatography-mass spectrometry; GSSG, glutathione disulfide; HIE, hypoxic-ischemic encephalopathy; LC-MS/MS, liquid chromatography-tandem mass spectrometry; LC-TOF-MS, liquid chromatography time of flight mass spectrometry; NMR, nuclear magnetic resonance; ROSC, return of spontaneous circulation; UPLC-MS/MS, ultra-performance liquid chromatography-tandem mass spectrometry; UPLC-QTOF-MS, ultra-performance liquid chromatography-quadrupole time of flight mass spectrometry.

**Table 2:** Metabolomics studies performed on human infants.

Reference	Population	Sample Groups	Biofluid	Method	Biomarkers Findings
Chu (2006) [44]	37	PA (with HIE or PND) vs. PA (without HIE) and controls	Urine	GC-MS	↑ Ethylmalonate, 3-hydroxy-3-methylglutarate, 2-hydroxy-glutarate, 2-oxo-glutarate
		PA (without HIE) vs. PA (with HIE or PND) and controls			↑ Glutarate, methylmalonate, 3-hydroxybutyrate, orotate
Longini (2015) [45]	14	PA vs. controls	Urine	^1^H NMR	↑ Lactate, glucose, trimethylamine-N-oxide, threonine and 3-hydroxyisovalerate ↓ Acetate, succinate, dimethylamine, citrate, dimethylglycine, creatine, creatinine, betaine, cis-aconitate, urea, formate
Noto (2016) [46]	12	Post-TH (different time points) vs. baseline	Urine	GC-MS	↑ Lactose, citrate, galactose, 4-hydroxyproline ↓ Lactate, taurine, lysine, mannitol, oxalate, fructose, N-acetylglucosamine
Locci (2018) [47]	26	HIE at birth vs. healthy controls at birth	Urine	^1^H NMR	↑ Lactate, myoinositol, betaine, taurine ↓ Citrate, acetone, dimethylamine, glutamine, succinate, pyruvate, α-ketoglutarate, N-acetyl groups, acetate, arginine
		HIE at day 3 vs. HIE at birth			↑ Creatine/creatinine, citrate, N,N-dimethylglycine, dimethylamine, cis-aconitate, 3-aminoisobutyrate, galactose, lactose, glutamine, α-ketoglutarate, glucose, N-acetyl groups ↓ Myoinositol, betaine, 1-methyl-nicotinammide, lactate, choline/phosphocholine, taurine, arginine, hypoxanthine
		HIE at day 30 vs. HIE at birth			↑ Citrate, betaine, dimethylamine, glutamine, pyruvate, α-ketoglutarate, galactose, lactose, formate, succinate, N-acetyl groups, N,N-dimethylglycine, cis-aconitate ↓ Myoinositol, lactate, choline/phosphocholine, 1-methyl-nicotinammide, arginine, acetate, hypoxanthine
Walsh (2012) [48]	142	PA vs. controls	Cord blood serum	LC-MS/MS	↑ Acylcarnitines, leucine
		HIE vs. controls			↑ Acylcarnitines, alanine, asparagine, isoleucine, leucine, methionine, phenylalanine, proline, tyrosine, valine
		Asphyxia vs. controls			↑ Glycerophospholipids, taurine
Reinke (2012) [49]	118	PA vs. controls	Cord blood serum	^1^H NMR	↑ 3-Hydroxybutyrate, acetone, alanine, betaine, choline, creatine, creatinine, glucose, glycerol, isoleucine, lactate, leucine, myoinositol, phosphocholine, phenylalanine, pyruvate, succinate, valine
		HIE vs. controls			↑ Methionine, alanine, choline, creatine, glycerol, isoleucine, lactate, leucine, myoinositol, phenylalanine, pyruvate, succinate, valine
		Severe HIE vs. controls			↑ Alanine, choline, creatinine, glycerol, lactate, succinate
Ahearne (2016) [50]	36	Severe outcome	Cord blood serum	^1^H NMR	Cord blood metabolite index (succinate•glycerol/3-hydroxybutyrate•phosphocholine) > 2.4 (sensitivity 80%, specificity of 100%)
		Normal outcome			Cord blood metabolite index (succinate•glycerol/3-hydroxybutyrate•phosphocholine) < 0.13 (sensitivity 65%, specificity of 91%)
Sánchez-Illana (2017) [51]	80	48 h after first dose of topiramate vs. controls	Cord blood plasma	GC-(EI)-Q-MS	↑ Pyruvate, lactate ↓ Acetoacetate, 3-hydroxybutyrate
		From birth to 72 h after first dose of topiramate			↑ Malate ↓ Lactate, pyruvate, 3-hydroxybutyrate
El-Farghali (2018) [52]	65	Asphyxia and HIE vs. controls	Cord blood dried spot	UPLC-MS	↑ Alanine, valine, phenylalanine, leucine, methionine, C0-carnitine, C10-carnitine, C2-carnitine, C4-carnitine, C5-carnitine, C8-carnitine, C18-carnitine
					↓ Citrulline/phenylalanine, histidine, ornithine, arginine, C3-carnitine, C14-carnitine, C16-carnitine

GC-(EI)-Q-MS, gas chromatography-electron ionization-quadrupole mass spectrometry; GC-MS, gas chromatography-mass spectrometry; HIE, hypoxic-ischemic encephalopathy; LC-MS/MS, liquid chromatography-tandem mass spectrometry; NMR, nuclear magnetic resonance; PA, perinatal asphyxia; PND, perinatal death; UPLC-MS, ultra-performance liquid chromatography-mass spectrometry.

**Table 3:** Potential biomarkers of PA in animal and human studies.

Metabolite	Sample	Reference
Lactate	Plasma, urine, cord blood, CSF, brain tissue, ocular tissues	[25,26,29,30,32,33,35,38,39,40,41,42,45,46,47,49,51]
Succinate	Plasma, urine, cord blood, brain tissue	[29,30,37,38,40,41,42,43,45,47,49,50]
Alanine	Plasma, urine, cord blood, CSF, brain tissue	[25,26,29,30,37,38,39,40,48,49,52]
BCAA (Ile, Leu, Val)	Plasma, urine, cord blood, CSF, brain tissue	[25,26,29,30,31,39,40,41,48,49,52]
Choline	Plasma, urine, cord blood, CSF, brain tissue	[25,26,29,31,32,33,40,43,47,49]
Glucose	Plasma, urine, cord blood, CSF, brain tissue	[25,35,37,38,40,41,45,47,49]
Creatinine	Plasma, urine, cord blood	[36,38,40,41,45,47,49]
Glutamine	Plasma, urine, brain tissue	[27,28,29,31,38,40,47]
Taurine	Urine, cord blood, brain tissue	[26,27,28,29,46,47,48]
Citrate	Urine, cord blood	[38,39,42,45,46,47]
Fumarate	Plasma, urine, cord blood, brain tissue	[29,30,39,40,42,43]
Malate	Plasma, cord blood, brain tissue, ocular tissues	[29,35,40,41,42,51]
Myoinositol	Plasma, urine, cord blood	[40,41,42,43,47,49]
Pyruvate	Plasma, urine, cord blood, ocular tissues	[35,38,40,47,49,51]
3-hydroxybutyrate	Plasma, urine, cord blood, CSF	[25,44,49,50,51]
Acetate	Plasma, urine, cord blood, brain tissue	[26,29,40,45,47]
Glutamate	Plasma, urine, brain tissue	[26,30,41,42,43]
Hypoxanthine	Plasma, cord blood, CSF	[25,31,32,40,47]
Phenylalanine	Urine, cord blood, CSF	[25,38,48,49,52]
Acylcarnitines	Plasma, cord blood	[30,31,48,52]
Betaine	Urine, cord blood	[39,45,47,49]
Creatine	Urine, cord blood	[39,45,47,49]
Formate	Urine, brain tissue	[29,40,45,47]
Glycine	Plasma, urine, cord blood	[30,38,40,43]
Phosphocholine	Urine, cord blood, brain tissue	[29,47,49,50]
Phosphocreatine	Brain tissue	[26,27,28,29]
α-Ketoglutarate	Plasma, urine, ocular tissues	[30,35,47]
Acetoacetate	Urine, cord blood	[37,38,51]
Arachidonate	Cord blood	[41,42,43]
Arginine	Urine, brain tissue	[26,47,52]
Dimethylamine	Urine	[37,45,47]
Glycerol	Cord blood	[41,49,50]
Methionine	Cord blood	[48,49,52]
Tyrosine	Cord blood CSF, brain tissue	[25,26,48]
Urea	Urine	[36,38,45]

BCAA, Branched Chain Amino Acids.
